# Battle of the Mondrians: Investigating the Role of Unpredictability in Continuous Flash Suppression

**DOI:** 10.1177/2041669518792930

**Published:** 2018-08-22

**Authors:** Shui’Er Han, David Alais, Randolph Blake

**Affiliations:** School of Psychology, The University of Sydney, New South Wales, Australia; Department of Psychology, Vanderbilt University, Nashville, TN, USA

**Keywords:** continuous flash suppression, binocular rivalry, perceptual bistability, visual perception

## Abstract

In continuous flash suppression (CFS), a dynamic sequence of Mondrian patterns presented to one eye suppresses a static target in the other eye for several seconds at a time. Its effectiveness has been linked to low-level properties such as spatial frequency and orientation, but the role of higher order influences remains unstudied. Here, using a tracking paradigm, we asked if the spatial and temporal predictability of the Mondrian sequence affects CFS dynamics. Predictable temporal sequences were regularly updated every 100 ms or modulated sinusoidally in pixel luminance at 2 Hz. Unpredictable temporal sequences were irregularly updated or had stochastic pixel luminance changes across time. To vary spatial predictability, sequences were either updated with different Mondrian patterns or had a fixed spatial pattern. We found a modest effect of spatial uncertainty when the target modulation was low (0.125 Hz) but not temporal uncertainty, which had no significant effects regardless of target modulation. Similar results were obtained when we pitted the standard Mondrian sequence against sequences with a fixed spatial pattern and temporally low-pass filtered sequences in a binocular rivalry paradigm. Thus, not only was the effect of information predictability was modest and spatial, but it was also dependent on the presence of higher temporal frequencies. Together, the results demonstrate the significance of low-level properties in affecting CFS dynamics and the possible involvement of pattern structure masking in CFS.

## Introduction

Psychologists have long been intrigued by the possibility that important aspects of perception and cognition could transpire outside of awareness (Driver & Mattingley, 1998; Pillai, 1939; Sidis, 1898; Vuilleumier et al., 2002). To chart the boundaries of processing outside of awareness, those of us who study perception exploit phenomena in which ordinarily detectable sensory stimuli are rendered undetectable. In the case of vision, there are a number of phenomena that entail just this characteristic and, thus, provide us with psychophysical tools for rendering the ordinarily visible invisible (Breitmeyer, 2014; Kim & Blake, 2005). Among those procedures for dissociating seeing and awareness are visual masking (Breitmeyer & Ogmen, 2000), motion-induced blindness (Bonneh, Cooperman, & Sagi, 2001), inattentional blindness (Neisser & Becklen, 1975) and binocular rivalry (Alais, 2012; Blake, 2001).

Twelve years ago, two research groups (Fang & He, 2005; Tsuchiya & Koch, 2005) independently described a particularly potent means for rendering an interesting, complex monocularly viewed stimulus invisible for many seconds at a time. Unlike binocular rivalry, this variant of interocular suppression entails presenting an animation consisting of rapidly changing series of geometric figures to one eye while simultaneously presenting a target stimulus to the corresponding retinal area of the other eye. In the version created by Fang and He, the successive animation frames comprised a random array of tiny squares, while the version created by Tsuchiya and Koch comprised successively presented coloured rectangles (a.k.a. *Mondrians*). With either configuration, the result was almost always immediate and relatively prolonged suppression of the target by the dynamic series of images, leading Tsuchiya and Koch to dub the technique continuous flash suppression (CFS). This compelling form of interocular suppression quickly caught the imagination of vision scientists interested in perceptual processing outside of awareness, spawning a slew of psychophysical papers utilising CFS to address that topic (see reviews by Gayet, Van der Stigchel, & Paffen, 2014; Sterzer, Stein, Ludwig, Rothkirch, & Hesselmann, 2014; Yang, Brascamp, Kang, & Blake, 2014). In addition, human brain imaging studies have deployed CFS to study the mode of action of CFS on neural activity within visual cortex (Watanabe et al., 2011; Yamashiro et al., 2014; Yuval-Greenberg & Heeger, 2013).

One lesson growing out of all of this work is an appreciation that CFS does not work with equal effectiveness for all participants (e.g., see the series of studies by Sklar et al., 2012, where results from up to 50% of participants had to be omitted because of insufficient depth of CFS suppression). As Vadillo, Konstantinidis, and Shanks (2016) have argued, this kind of rejection of false-positive data raises statistical issues that can compromise conclusions from studies on unconscious processing. One way to minimise the problem is to use calibration procedures to establish CFS and target parameters that produce uniform depth of suppression among all participants (see Han & Alais, 2018; Hesselmann, Darcy, Rothkirch, & Sterzer, in press). At the same time, it is important to establish the boundary conditions governing the maximum effectiveness of CFS. To date, the majority of investigations utilising CFS have employed variants of the configurations introduced by Fang and He (2005) and Tsuchiya and Koch (2005), namely a dynamic mask consisting of an ever-changing stream of texture arrays flashed one after the other at a rate of 10 frames/second (10 Hz).

On the belief that good workers benefit from understanding their tools, we and others have endeavoured to explore factors that contribute to the effectiveness of CFS. Thus, it is now known that CFS potency varies with the spatial frequency of the masker (Willenbockel, Lepore, Nguyen, Bouthillier, & Gosselin, 2012; Yang & Blake, 2012), the contrast of the masker (Han, Lunghi, & Alais, 2016; Ledgeway, McGraw & Thompson, 2013; Tsuchiya & Koch, 2005), the update rate of the masker (Zhu, Drewes, & Melcher, 2016) and the temporal frequency content of the masker (Han, Blake & Alais, 2018; Han et al., 2016). Previous CFS-masked priming experiments also reported shorter response times to tool probes when elongated prime stimuli were used (Almeida et al., 2014), but these results might have been partially influenced by image leakages in anaglyph glasses and response time variability to the different probe stimuli (Hesselmann, Darcy, Rothkirch, & Sterzer, 2018). Another factor impacting CFS potency is repeated testing: The strength of suppression tends to wane with repeated testing, as evidenced by the propensity for a target to breach CFS suppression earlier and earlier over repeated trials (Blake, Goodman, Tomarken, & Kim, in preparation; Ludwig, Sterzer, Kathmann, Franz, & Hesselmann, 2013). This latter observation led us to wonder whether the predictability of the dynamics of the CFS sequence might have an impact on the strength of CFS, and this is the idea tested in the experiments described in this article.

The present study was inspired by the concept of entropy (Shannon, 1948), as it has been applied to other aspects of visual perception (e.g., see Gilden, Hiris, & Blake, 1995; Lee, Blake, & Lee, 2009). Expressed mathematically, entropy is a quantity related to the Poisson rate parameter associated with a given time series. In simple terms, entropy is an expression of predictability, where successive events unfolding regularly over time have low entropy, whereas successive events occurring irregularly have higher values of entropy. The entropy value would depend on the number of alternative random event states and their relative probabilities, but as rule of thumb, entropy is inversely proportional to predictability. Viewed within an entropy framework, the commonly used CFS masking stimulus of a series of random spatial Mondrian patterns presented at a rate of 10 Hz has low entropy in the temporal domain, as changes will occur predictably every 100 ms, and high entropy in the spatial domain, as each new Mondrian pattern in the sequence is random and unrelated to its predecessor. In this study, we have examined the consequences of manipulating temporal and spatial entropy on the potency of CFS, as assessed by its suppression of a contralateral bullseye target over a 1-minute viewing period. This allowed us to measure the initial strength of suppression as typically recorded in breaking-CFS studies (Jiang, Costello, & He, 2007; Moors, Wagemans, & De-Wit, 2014; Yang, Zald, & Blake, 2007) and to characterise masker effectiveness over a longer period of time. All experiments described in this study adopted a dominance tracking paradigm that is used in previous CFS (Tsuchiya & Koch, 2005; Yamashiro et al., 2014) and rivalry studies (Alais, Cass, Shea, & Blake, 2010; Andrews & Blakemore, 2002; Brascamp, van Eee, Noest, Jacobs, & van den Berg, 2006; Dieter, Melnick, & Tadin, 2015; Kang, 2009; Parker & Alais, 2007).

## Experiments 1 and 2: The Regularity of Pattern Changes

**Figure 1.**
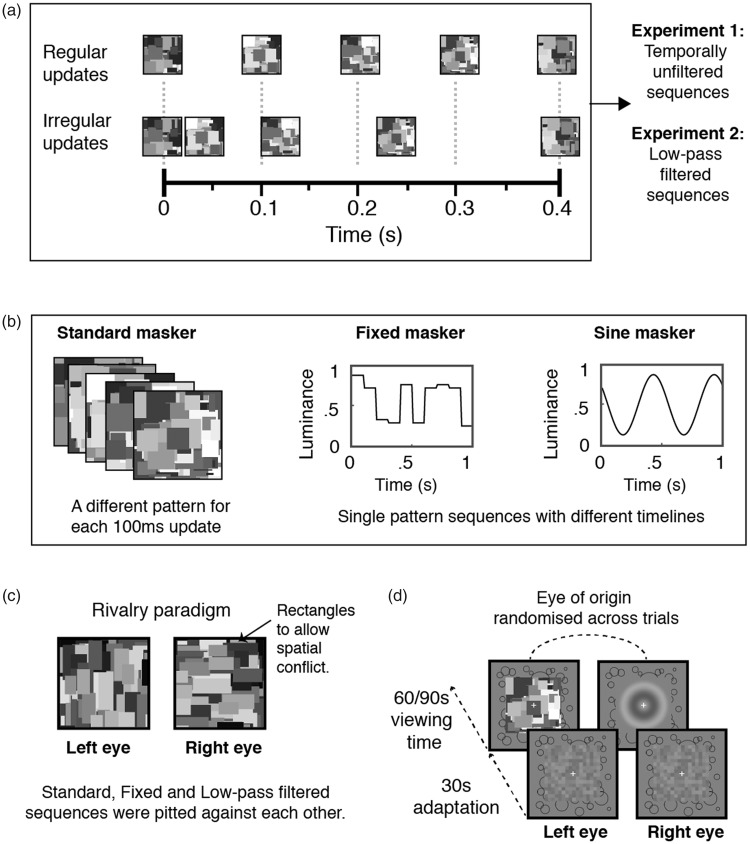
Stimuli used in this study. (a) Presentation schedules used in Experiments 1 and 2. Patterns were updated regularly every 100 ms or at irregular intervals (i.e., 40 ms, 80 ms, 120 ms and 160 ms, averaging 100 ms). The original temporally unfiltered sequences were used in Experiment 1, whereas the sequences were temporally low-pass filtered in Experiment 2. (b) Maskers used in Experiments 3 and 4. Three types of maskers were used to produce different degrees of spatial and temporal entropy. The standard masker was updated with a different pattern every 100 ms, meaning that it had random luminance and spatial pattern profiles (i.e., high spatial and temporal entropy). The fixed masker was a single pattern sequence that had the same temporal timeline as the standard masker, and as such, had low spatial entropy. The sine masker was also a single pattern sequence, with the exception that it modulated regularly (and more predictably than the fixed masker) at 2 Hz. (c) Maskers used in Experiment 5. The Standard and Fixed sequences from Experiments 3 and 4 were pitted against temporally low-pass filtered Mondrian sequences in rivalry paradigm. The patterns were composed of rectangles instead of squares. (d) Stimulus presentation for each trial. For Experiments 1 to 4, each trial begins with a 30-s adaptation phase of spatial noise composed of 0.5 by 0.5 squares, followed by the dichoptic presentation of a Mondrian sequence and a 1 cycle per degree, grey scale bullseye target. For Experiment 5, however, the target was replaced by a Mondrian sequence. Participants were asked to track the appearance of the target (Experiments 1–4) or the perceptual dominance of either Mondrian sequence (Experiment 5). All visual stimuli were enclosed with a fusion frame, which is shown to both eyes to encourage binocular fusion. The presentation of the visual stimuli was fixed within a trial but randomised between eyes across trials.

These experiments asked if the regularity of Mondrian pattern changes affects the dynamics of CFS. As shown in Figure 1(a), two types of temporal schedules were compared in Experiment 1: Regular pattern updates every 100 ms or irregular pattern update periods varying around an average of 100 ms. These temporal schedules were then temporally low-pass filtered (<4 Hz) and compared in Experiment 2. This was to ascertain the influence of temporal irregularity, as the irregular update schedule contained longer periods of stationary patterns that could potentially weaken suppression through increased neural adaptation (Alais et al., 2010). By low-pass filtering the temporal schedules, we retained the occurrence of pattern changes but standardised the dominant temporal frequency content (Han et al., 2016, 2018), while producing a continuously varying pixel luminance timeline.

### Materials and Methods

#### Participants

Recruitment and testing were conducted in the University of Sydney and Vanderbilt University. As we were interested in evaluating the practical usefulness of entropy in providing effective interocular suppression, we determined our sample size based on previous work, where the estimated effect sizes with 7 to 11 participants were 0.55 to 0.84 using partial eta-squared (Han et al., 2016). Thus, a total of 11 participants were recruited for Experiment 1, of which 8 of them (5 females), including two of the authors (R. B. and S. H.), also took part in Experiment 2. Two other participants (one female) only participated in Experiment 2, giving a total of 10 participants in Experiment 2. All participants had normal or corrected-to-normal eyesight, and tested normal for stereovision, with the Randot Stereo Test or the Fly Stereo Acuity Test. Experiments accorded with the Declaration of Helsinki and were approved by the institutional review board of the University of Sydney and Vanderbilt University. All participants provided informed consent and were reimbursed for their time.

#### Visual stimuli

Masker stimuli were dynamic sequences of randomly generated 4° by 4° grey scale Mondrian pattern images composed of squares ranging from 0.6° to 1.2° in length. These sequences were updated in a temporally regular or irregular manner. Temporally regular sequences in Experiment 1 were updated every 100 ms, whereas irregular sequences were updated at intervals randomly selected from a pre-determined set of temporal intervals of 40, 80, 120 and 160 ms (averaging 100 ms; Figure 1(a)). For Experiment 2, these sequences were temporally low-pass filtered in frequency space. A three-dimensional fast Fourier transform was first performed on each pattern sequence, followed by the application of a low-pass temporal filter (<4 Hz) to the amplitude spectrum in the temporal dimension, with no manipulation of the orthogonal spatial dimensions. The filtered spectral components were then back-transformed to image space. In each trial, the Mondrian sequence lasted 60 s, had a root mean square (RMS) contrast of approximately 20% and was normalised to mean luminance.

The target was a two-dimensional bullseye (Figure 1(b)), generated by computing a concentric sinusoidal grating with a spatial frequency of 1 cycle per degree and a radius of 3.3° visual angle. To avoid afterimages in the suppressed eye (Gilroy & Blake, 2005), the target was phase modulated at a rate of 0.125 Hz throughout the duration of dichoptic stimulation for each trial. During the experiment, the target was presented at 30% of maximum contrast, and the masker was set to full RMS contrast. These values were subjectively adjusted for two participants, who reported no target breakthrough with the aforementioned settings. Higher bullseye contrasts were used for these participants as a result. To encourage stable fusion, a fusion frame with an internal width of 4° and an external length of 6.5° was used to enclose all visual stimuli. Visual stimuli were displayed to participants using a ViewSonic PS225f or Triniton Dell CRT monitor (resolution 1024 × 768 pixels for both) at the University of Sydney, and a Sony GDM-F90 CRT monitor (resolution 800 × 600 pixels) was used at Vanderbilt University. All visual displays were gamma-corrected and had a screen rate of 100 Hz.

#### Procedure

Participants viewed the stimuli through a mirror stereoscope. To ensure that the dichoptic images were fused, each individual first completed a calibration task where the perceived spatial positions of two 4° by 4° by 0.3° thick square frames were aligned using key presses to nudge the location of one of them vertically or horizontally. Following that, participants commenced the CFS task. Each trial began with a prompt to initiate stimulus presentation with a key press, after which participants were adapted to a 30 s sequence of noise images (composed of 0.5° by 0.5° squares, normalised to mean luminance: see Figure 1). Post-adaptation, the masker was presented at the same contrast as in adaptation, whereas the target’s contrast was gradually increased to the preset target contrast over a period of 800 ms. Both target and masker were presented on the screen for 60 s, during which participants were asked to press a key when any part of the target became visible and to hold it down for as long as it remained visible. Each trial thus provided the respective dominance durations of the Mondrian masker and the target, and the number of target breakthroughs. All participants were instructed to fixate on the central cross throughout the duration of each trial and were given ample practice to familiarise with the task demands. To avoid inter-trial effects between the experimental conditions, the effects of irregular and regular pattern update schedules were tested in counterbalanced blocks. Each block consisted of four trials that were counterbalanced for the eye of presentation (i.e., each eye received the masker two times in each block). Each individual thus completed a total of eight trials (four for each eye) for each condition.

#### Analysis

**Figure 2.**
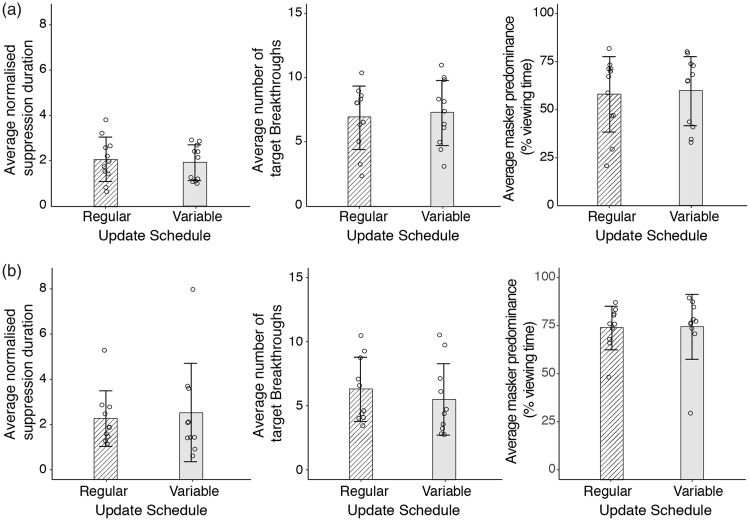
Results of Experiments 1 and 2. From left to right, the three panels show results for the average normalised time to the first target breakthrough, average number of target breakthroughs and average masker predominance. (a) Experiment 1: results for unfiltered Mondrians. The distributions of individual data points, the median and the median absolute deviation (denoted by error bars) were plotted. None of the dependent measures was significantly influenced by the regularity of pattern update, as the data sets for both types of pattern update had comparable medians and considerable variability. (b) Experiment 2: results for low-pass filtered Mondrians. Similar to Experiment 1, the data sets for both types of update schedules have considerable variability and comparable medians and do not differ on any of the dependent measures. These results indicate that the regularity of pattern update has no effect on CFS dynamics, and that the lack of significant effects is not confounded by neural adaptation, which conceivably could have played a stronger role in the temporally unfiltered, irregular update schedule.

Three dependent measures were computed from each individual’s data set, namely, the time to first target breakthrough, the average number of target breakthroughs and masker predominance. Prior to computing the dependent measures, the dominance durations of the Mondrian masker were first normalised to the overall average duration. We then extracted the normalised time to first target breakthrough (henceforth referred to as normalised suppression duration) and computed the average for each condition. This was synonymous with the time to visibility measured in previous breaking-CFS studies (e.g., Jiang et al., 2007) and reflected the strength of the initial suppression. The average number of target breakthroughs and average masker predominance reflected the overall masker effectiveness over the course of the trial. For each condition, we computed the average number of target breakthroughs and averaged masker predominance, defined as the total proportion of viewing time in a trial where the Mondrian was perceptually dominant (see also Brascamp, Klink, & Levelt, 2015). Individual data points were plotted for all dependent measures and were also summarised with the median and median absolute deviation of the between-subjects data points (see Figure 2). Pairwise comparisons were conducted with the non-parametric Wilcoxon signed-rank test computed using the Hollander and Wolfe (1973) method in the statistical program R (R Core Team, 2017). This method uses the parameter *V*, which is obtained by first computing the difference between the data compared, and then summing the ranks assigned to positive differences.

### Results

The results of Experiments 1 and 2 are summarised in Figure 2(a) and (b), respectively. Individual data points, each representing the average normalised suppression duration (left panels of Figure 2), average number of target breakthroughs or average masker predominance of each participant, are plotted for both experiments. Given the variability in individual data points, the median was used to represent the central tendencies of the different dependent measures. As shown in Figure 2(a), manipulating the temporal regularity of pattern updates in Experiment 1 produced comparable normalised suppression durations, masker predominance and number of target breakthroughs. The same conclusions are also reflected statistically, where we obtained no significant differences in normalised suppression durations, *V* = 21, *p* = .32, masker predominance, *V* = 43, *p* = .41 and number of target breakthroughs, *V* = 35.5, *p* = .41 (corrected for continuity).

Similarly, in Experiment 2, low-pass filtered regular and irregular pattern sequences produced comparable normalised suppression durations, masker predominance and target breakthrough frequencies (Figure 2(b)). Despite controlling for differences in temporal frequency content, pattern update regularity did not significantly affect the initial breakthrough time of CFS, *V* = 33, *p* = .63. In addition, update regularity did not have a significant influence on the overall masker effectiveness over prolonged periods of time, *V* = 37, *p* = .38. Similarly, the number of target breakthroughs was also not significantly different between the two types of pattern sequences, *V* = 10, *p* = .16 (corrected for continuity).

### Discussion for Experiments 1 and 2

In Experiments 1 and 2, we varied the regularity of pattern updates to learn whether difference in temporal entropy has an effect on CFS effectiveness. Irregular and regular update schedules were compared in Experiment 1, and the comparison was repeated with temporally low-pass filtered schedules in Experiment 2. Three dependent measures were collected, namely, the time to first target breakthrough, number of target breakthroughs and masker predominance. Initial breakthrough times were synonymous with previous breaking-CFS studies (e.g., Jiang et al., 2007) and reflected the effectiveness of the initial suppression. On the other hand, the average number of target breakthroughs and masker predominance tapped into the effectiveness of suppression over a prolonged period of time. Our results showed that in neither experiment did pattern update regularity have a significant effect on any of the three dependent measures.

**Figure 3.**
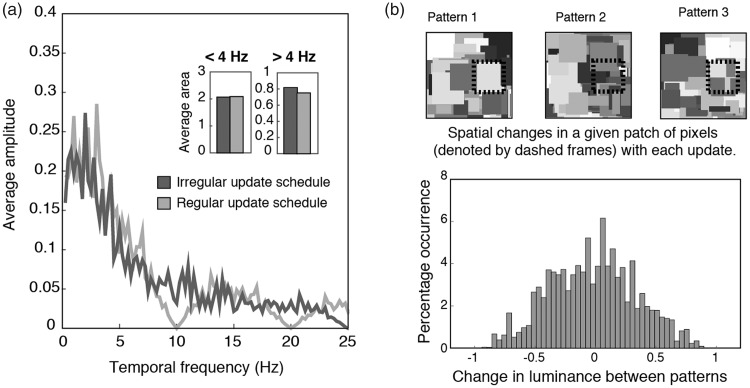
Properties of the Mondrian maskers. (a) The effect of pattern update regularity on the temporal frequency spectrum of the Mondrian masker. Amplitude spectra shown were obtained by conducting a one-dimensional fast Fourier transform on pixel timelines of the irregular and regular Mondrian sequences. Both types of sequences have a characteristic 1/f profile, which were comparable in low (<4 Hz) and high (>4 Hz) frequency content, assessed with the average area under the curve estimated from 10 independently drawn pixel timelines. (b) Spatial and temporal entropy in the Mondrian masker. As shown in the upper panel, spatial changes between patterns were unpredictable, varying in luminance, size and configuration for a given patch of pixels in the Mondrian masker. On the other hand, temporal entropy (lower panel) was limited by the normal distribution of luminance changes between pattern updates, as most pixel changes were of a smaller magnitude.

One possible reason for the absence of an effect of entropy was that the irregular and regular pattern update schedules had different temporal frequency content. We doubt this was the case, as Fourier analyses revealed comparable temporal frequency content between the two types of schedules in Experiment 1 (Figure 3(a)). Moreover, similar results were obtained when the temporally low-pass filtered maskers were used in Experiment 2. Instead, it seemed more likely that our manipulation of entropy was inadequate, producing perhaps a subtle change in predictability that was easily obscured by subjective sources of variability such as participant decisional criteria (Yang et al., 2014). Figure 3(b) illustrates this point. Each pattern update in the Mondrian masker was accompanied by changes in spatiotemporal information. With every new pattern, spatial elements of the Mondrian masker vary in location, contour shapes and size (Figure 3(b), upper panel). Temporal changes were also stochastic, though more predictable than spatial changes as the differences in luminance between patterns tended to be of a smaller magnitude (Figure 3(b), lower panel). By influencing the temporal dimension with variations in the update schedules in Experiments 1 and 2, we ignored a potentially important source of information entropy. Instead, we varied a dimension that had more predictable outcomes. In the following experiments, we adopted a more comprehensive definition of entropy that involves both the spatial and temporal domains.

## Experiments 3 and 4: The Effect of Spatiotemporal Entropy

Experiments 1 and 2 failed to yield any conclusive evidence regarding the effect of temporal regularity on the dynamics of CFS, leading us to wonder whether our manipulation of entropy was adequate. We thus defined predictability more comprehensively in Experiments 3 and 4, this time focusing on changes in spatial patterns and pixel luminance. Three types of maskers were compared, namely, a standard 10 Hz Mondrian masker (*Standard*), a single Mondrian pattern that modulated in luminance sinusoidally at 2 Hz (*Sine*) or had pixel luminance timelines extracted from a regular Mondrian (*Fixed*). Of these maskers, the standard masker had the highest spatial and temporal uncertainty and therefore the highest entropy in both dimensions. The sine masker, given the use of the single pattern and periodic changes in luminance, had the lowest spatial and temporal uncertainty and thus lowest entropy. The predictability of the fixed masker lay in between, as it had low spatial entropy but with the same stochastic changes in luminance as the standard masker. For the target stimulus, we chose a phase modulation rate of 0.125 Hz in Experiment 3 and a rate of 4 Hz in Experiment 4. These values were chosen because the slower target rate was a better match to temporal frequency content of all three types of maskers (i.e., 2 Hz sine maskers and 1/f spectrum for the standard and fixed maskers), allowing us to test if entropy provides a suppressive advantage beyond that of temporal frequency selectivity (Han & Alais, 2018). Given that pixel luminance changes between Mondrian patterns tended to cluster around smaller magnitudes in the standard masker, as shown in the lower panel of Figure 3(b), we expected the manipulation of spatial entropy to have a larger effect on suppression, for both temporally similar and dissimilar targets.

### Materials and Methods

#### Participants

Recruitment and testing were conducted in the University of Sydney and Vanderbilt University. The required sample size was determined using the same criteria as Experiments 1 and 2. Five participants (four females), including author S. H., participated in Experiments 3 and 4. Six other participants (three females) only took part in Experiment 3, and another five participants (three females) only participated in Experiment 4. All participants had normal or corrected-to-normal eyesight, and tested normal for stereovision, with the Randot Stereo Test or the Fly Stereo Acuity Test. Experiments accorded with the Declaration of Helsinki and were approved by the institutional review boards of the University of Sydney and of Vanderbilt University. All participants provided informed consent and were reimbursed for their time.

#### Visual stimuli

Three types of masker stimuli were used in Experiments 3 and 4. They were dynamic sequences of Mondrian patterns (Standard) that were updated every 100 ms, single Mondrian patterns that modulated in luminance sinusoidally at 2 Hz (Sine) or single Mondrian patterns that had the same pixel luminance timeline as the Standard Mondrian sequence (Fixed). All Mondrian patterns used in these experiments were grey scale, 4° by 4° in size and composed of squares ranging from 0.6° to 1.2° in length. As in the previous experiments, each sequence lasted for 60 s in each trial, had a RMS contrast of approximately 20% and was normalised to mean luminance. The bullseye target from Experiments 1 and 2 was used in Experiments 3 and 4, with the exception that the target was presented at a lower contrast (20% instead of 30% of maximum RMS contrast) and phase-modulated at a rate of 4 Hz in Experiment 4. All visual stimuli were enclosed with frames measuring of 4° internally and 6.5° externally to encourage stable fusion. The same display apparatuses from Experiments 1 and 2 were used to present the visual stimuli.

#### Procedure

The same procedures from Experiments 1 and 2 were used in Experiments 3 and 4.

#### Analysis

The data sets were prepared in the same manner as in Experiments 1 and 2. Similarly, non-parametric statistical approaches such as Wilcoxon signed-rank tests and Friedman tests were used to assess pairwise comparisons and main effects, respectively.

### Results

**Figure 4.**
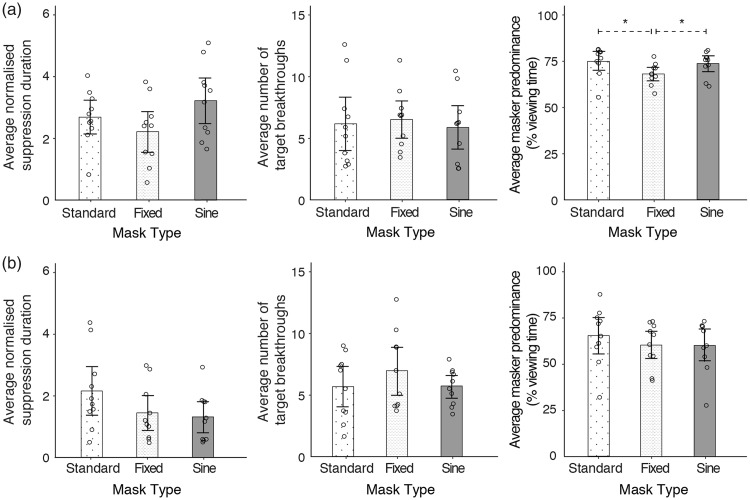
Results of Experiments 3 and 4. From left to right, the three panels show results for the normalised time to the first target breakthrough, average number of target breakthrough and masker predominance. (a) Experiment 3: the effect of spatial and temporal entropy on the suppression of a 0.125 Hz target. The bar graphs represent the distributions of data points, the median and the median absolute deviation (denoted by error bars). The average number of target breakthroughs and normalised suppression durations was not significantly influenced by the regularity of pattern update, as the data sets for both types of pattern update had comparable medians and considerable variability. On the other hand, the predominance of the fixed masker was significantly lower than that of the sine and standard maskers. As there was no significant difference between the sine and standard maskers, the predominance results demonstrate the importance of spatial entropy in stepped timelines. (b) Experiment 4: the effect of spatial and temporal entropy on the suppression of a 4 Hz target. Although the standard masker produced slightly longer normalised times to first target breakthrough and larger masker predominance than the other two maskers, none of the dependent measures was significantly affected by entropy. Error bars represent the median absolute deviation of individual data points.

The results of Experiments 3 and 4 are illustrated in Figure 4. As in the previous experiments, individual data points are plotted to show the average normalised suppression duration, average masker predominance and average number of target breakthroughs for each participant. Central tendencies of each dependent measure are also represented by the respective medians. As shown in Figure 4(a), fixed maskers produced lower masker predominance and normalised suppression durations than the sine and standard maskers. Normalised suppression durations were approximately 10% to 30% lower, and the fixed masker dominated approximately 11% less amount of viewing time than the other two maskers. The number of target breakthroughs, however, was comparable across all three maskers. These observations were assessed with the Friedman test, a non-parametric version of the one-way repeated-measures analysis of variance. The results reveal no significant main effects of masker type on normalised suppression durations and number of target breakthroughs, χ^2^(2) = 5, *p* = .08 and χ^2^(2) = 3, *p* = .22, respectively. However, masker type did affect masker predominance significantly, χ^2^(2) = 7.8, *p* = .02. The effect of masker type on masker predominance was assessed using Holm-Bonferroni corrected Wilcoxon signed-rank tests. We found that the presence of uncertain spatial information in the standard masker produced a significantly higher predominance than the fixed masker, *V* = 51, *p* = .03, but the effect seemed contingent on the use of the stepped timeline in the fixed masker. This was because the predominance of the sine and standard maskers were statistically no different, *V* = 32, *p* = .70. In addition, the sine masker had a significantly higher predominance than the fixed masker, *V* = 5, *p* = .02, even though the latter had a broader temporal frequency spectrum.

The results of Experiment 3 suggest a modest effect of spatial entropy on masker predominance, which we tested with a less compatible, 4 Hz target in Experiment 4. Figure 4(b) shows that the median masker predominance is approximately 7% higher for the standard masker than the other two maskers. However, compared with Experiment 3 (Figure 4(a)), the distribution of individual data points for masker predominance is noticeably more variable across all three maskers. For normalised suppression durations, suppression was approximately 40% longer when the standard masker was used. Compared with the sine and standard maskers, target breakthroughs were approximately 14% higher when the fixed masker was presented. None of these observations is statistically significant. Masker type did not affect the normalised suppression duration, χ^2^(2) = 3.8, *p* = .15, and had no significant effect on target breakthrough frequency, χ^2^(2) = 4.7, *p* = .10. Despite the presence of both spatial and temporal entropy in the standard masker, masker type had no significant effect on masker predominance, χ^2^(2) = 4.2, *p* = .12. Thus, the effect of spatial entropy appears to depend on the presence of compatible target or masker temporal frequency content.

### Discussion for Experiments 3 and 4

We had reasoned that our manipulation of entropy in Experiments 1 and 2 might have been too simplified in focusing only on the temporal dimension. Thus, in Experiments 3 and 4, we adopted a broader manipulation of entropy, taking into account the predictability of the spatial and temporal content. Three types of Mondrian maskers were used, namely, the standard 10 Hz Mondrian pattern sequences (Standard), single Mondrian pattern maskers with the same pixel luminance timeline as the standard Mondrian (Fixed) and single patterns that modulated sinusoidally in luminance at 2 Hz (Sine). To assess the extent of any possible effects of entropy, target rates that were similar to the maskers (0.125 Hz, Experiment 3) and dissimilar to the maskers (4 Hz, Experiment 4) were used. Because pixel luminance changes between Mondrian patterns tended to be of a smaller magnitude (Figure 3(c)), we expected spatial entropy to have a larger effect on suppression than temporal entropy, regardless of the target modulation rate. Although we did find a significantly larger predominance for the standard masker than the fixed masker in Experiment 3, performance was comparable between standard and sine maskers, indicating that the effect was limited to maskers with stepped timelines. This weak influence of entropy was further demonstrated in Experiment 4, where the use of the less compatible, 4 Hz target resulted in a null result for all three dependent measures. Together, the results of Experiments 3 and 4 did not support a substantial influence of entropy in CFS.

Differences in individual contrast sensitivity and decisional criteria in determining target visibility (Yang et al., 2014) might have contributed to the variability in the data, reducing statistical power as a result. Also, it is possible that the extent to which entropy affects CFS could be better estimated by measuring suppression depth or target contrast thresholds over time. However, we have observed that comparisons of contrast thresholds and suppression durations in CFS show a high correlation (Han & Alais, 2018), and we conclude it is more likely for weaker effects to be obscured by between-subject variability. Instead, lower level factors seemed to have a larger influence on CFS than entropy. To elaborate, periods of constant illumination in a masker with a stepped update schedule meant that the fixed and standard maskers were more susceptible to neural adaptation. As mentioned earlier, increasing neural adaptation weakens interocular suppression (Alais et al., 2010). Thus, any loss in suppressive strength over prolonged periods of time might have been partially compensated by the presence of spatial uncertainty in the standard masker. With a continuously varying luminance timeline, such compensation was not necessary for the sine masker, explaining why the standard and sine maskers were comparable in performance in Experiment 3. In addition, since the 4 Hz target in Experiment 4 was temporally less compatible to all three maskers, the failure to observe a significant effect of spatial entropy in Experiment 4 underscores the weak influence of spatial entropy and the importance of low-level factors such temporal frequency selectivity compared with spatial entropy.

Previous studies have suggested the involvement of adaptation (Tsuchiya & Koch, 2005) and feature selectivity in CFS (Han & Alais, 2018; Moors et al., 2014; Yang & Blake, 2012; but see Ananyev, Penney, & Hsieh, 2017), and it was not surprising that these factors might contribute to our results. More interestingly, was the nature in which spatial entropy contributed to CFS. As the effect of spatial entropy was limited to the use of stepped pattern presentation schedules, one possible mechanism was pattern structure masking. To expand on this point, masking effectiveness was reported to increase with shorter temporal intervals (Enns & Di Lollo, 2000) and sharp temporal onsets (Macknik, Martinez-Conde, & Haglund, 2000). The transient, unpredictable phase congruency changes in the standard masker fit this bill, suggesting that the effect of spatial entropy was probably dependent on the presence of higher temporal frequencies. In the absence of higher temporal frequencies (e.g., sine masker), spatial entropy was not particularly important in CFS and vice versa (e.g., fixed masker). Alternatively, the apparent link between spatial entropy and higher temporal frequencies might be a consequence of our CFS stimulus choices. In common with most CFS studies, our stimuli involved a high-contrast masker and a low-contrast target, resulting in a significant imbalance of stimulus strength between the two eyes. The imbalance in contrast has been shown to be crucial to CFS suppression (Han et al., 2016; Tsuchiya & Koch, 2005), and combined with other low-level influences such as feature selectivity, any contributions from entropy on the CFS process might have been limited by the strong existing suppression of the target. Thus, instead of a relationship between spatial entropy and temporal frequency content, the effect of spatial entropy might have been more easily revealed in the stepped schedules because of greater adaptation. Finally, a third possibility was that entropy had a weak influence on interocular suppression and would remain so regardless of the distribution of stimulus strength between the eyes. To tease apart these possibilities, we would have to eliminate the imbalance in contrast, and one way to do so would be to have the maskers compete directly in a binocular rivalry paradigm.

## Experiment 5: Boxing Match Between Mondrian Contenders

Experiments 3 and 4 revealed a measurable, albeit modest, influence of spatial entropy on CFS suppression strength that was limited to the use of stepped presentation schedules. As the stepped pattern presentations (i.e., fixed and standard maskers) had 1/f, broadband temporal frequency spectrums (cf. Figure 3 and Han et al., 2016), the results suggested a relationship between spatial entropy and the low energy, higher temporal frequency content. Here, we examined this relationship by pitting three pairings of standard, fixed and low-pass filtered ( < 4 Hz) Mondrian sequences against each other in a rivalry paradigm. By determining which sequence would be perceptually dominant, this approach allowed us to re-examine the effect of entropy in the absence of the contrast imbalance typically used in CFS studies (e.g., Tsuchiya & Koch, 2005; Yang & Blake, 2012). Second, evaluating the effectiveness of the maskers against each other was a more direct way of assessing entropy’s role in interocular suppression. Third, as the low-pass sequence retained spatial entropy, pitting the continuously varying low-pass filtered Mondrian sequence against the fixed and standard sequences allowed us to test the relationship between spatial entropy and higher temporal frequencies. To draw a light-hearted analogy, the rivalry task was colloquially referred to as a *boxing match*. Since there were three combinations of competing Mondrian sequences (i.e., standard vs. fixed, standard vs. low-pass and fixed vs. low-pass), a total of three matches were conducted in Experiment 5. Examples of the different Mondrian sequences are provided (see movies: *Vertical Standard, Vertical Fixed and Vertical Low-Pass*).

Perceptual judgments were only possible if the competing maskers were discriminable. We thus generated Mondrian pattern sequences composed of either horizontal or vertical rectangles. During the experiment, the orientation of each sequence was presented orthogonally to the competing sequence in the other eye. Assuming that the results in Experiments 3 and 4 did reflect pattern structure masking, we predicted that spatial entropy should have a larger effect on rivalling dynamics when it is paired with higher temporal frequency content. Thus, when pitted against either the low-pass or fixed sequence, the standard sequence was expected to dominate the boxing match. In contrast, rivalling the fixed and low-pass sequences were expected to produce a draw, since neither sequence contained *rapidly varying* pattern contours and shapes.

### Materials and Methods

#### Participants

Recruitment and testing were conducted at the University of Sydney and Vanderbilt University. The required sample size was determined using the same criteria as the previous experiments. Seven participants (five females) participated in Experiment 5. All participants had normal or corrected-to-normal eyesight, and tested normal for stereovision, with the Randot Stereo Test or the Fly Stereo Acuity Test. Experiments accorded with the Declaration of Helsinki and were approved by the institutional review board of Vanderbilt University. All participants provided informed consent and were reimbursed for their time.

#### Visual stimuli

Each Mondrian pattern was made up of horizontally or vertically oriented rectangles ranging from 0.47° to 0.70° in width and 1.41° to 2.34° in height. All patterns were grey scale and measured 4° by 4° in size. The standard and fixed Mondrian sequences were generated with the same procedure as Experiments 3 and 4, and the low-pass sequence was generated using the same low-pass filtering procedure used in Experiment 2. All Mondrian sequences were set to maximum contrast (RMS = 25%), normalised to mean luminance and were presented equally in both orientations. To produce binocular rivalry, we ensured there was always an interocular spatial conflict by presenting the vertical rectangles to one eye and the horizontal rectangles to the other. As in previous experiments, all visual stimuli were enclosed with frames measuring of 4° internally and 6.1° externally to encourage stable fusion. The same display apparatus from Vanderbilt University was used to present the visual stimuli.

#### Procedure

The procedures followed in Experiment 5 were largely similar to the previous experiments, with the exception that the rivalling stimuli were presented for 90 s instead of 60 s. Participants pressed the left arrow key while they saw a horizontal rivalling stimulus, and the right arrow key while they saw the vertically oriented stimulus. Each trial thus provided the respective dominance durations of each Mondrian sequence and the number of alternations. Periods of piecemeal rivalry were not recorded. As with the previous experiments, participants were instructed to maintain fixation on the centre cross throughout the duration of each trial and were given ample practice to familiarise with the task demands. To avoid inter-trial effects between the experimental conditions, each pair of competing Mondrian patterns was presented in counterbalanced blocks. The pairs presented were the standard and fixed sequences, the standard and low-pass sequences or the fixed and low-pass sequences. Each block consisted of four trials that were counterbalanced for the eye of presentation (i.e., each eye received each sequence twice in each block). Each individual thus completed a total of eight trials (four for each eye) for each condition.

#### Analysis

Each boxing match involved rivalling a specific pair of Mondrian sequences (e.g., low-pass vs. fixed or standard vs. fixed). Thus, the winning Mondrian sequence of a specific match was determined by its overall predominance and mean dominance duration across all trials. Prior to computing these dependent measures, we normalised the dominance durations of each Mondrian sequence to the average dominance duration of each match. As in the previous experiments, we determined a sequence’s predominance for each trial by computing the proportion of its dominance out of the total viewing time. The respective predominance values were then averaged across trials and compared against the competing sequence using the Wilcoxon signed-rank test. Similarly, mean dominance durations were computed for each trial and then averaged across trials for each competing sequence. To compare the dynamics of each match, the number of perceptual alternations of each match was averaged across trials and statistically assessed with the Friedman’s test. Finally, as the human sensitivity to horizontal and vertical lengths has been reported to differ (Gottsdanker & Tietz, 1992), we compared the predominance and mean dominance durations of vertically and horizontally oriented sequences.

### Results

**Figure 5.**
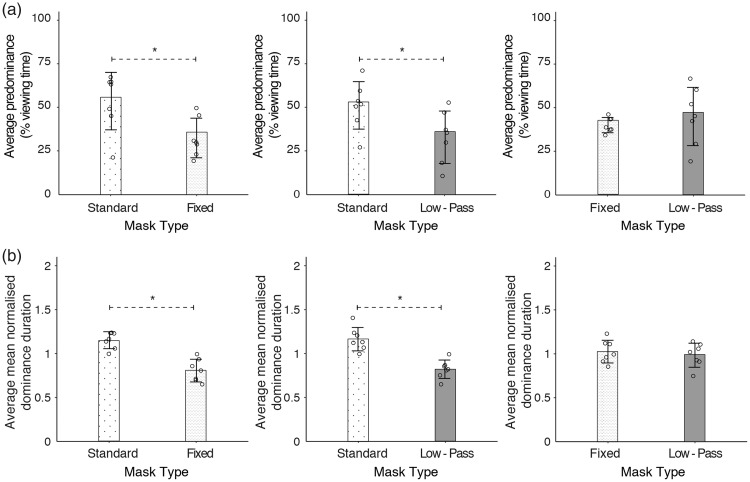
Results for the predominance and mean dominance duration in Experiment 5. From the left panel, the boxing match results for each rivalling pair. Rivalling pairs presented were the standard and fixed sequences, the standard and low-pass sequences and the fixed and low-pass sequences. (a) Predominance results were summarised as bar graphs, which represent the distributions of data points, the median and the median absolute deviation (denoted by error bars). The standard sequence had a significantly higher predominance than the fixed and low-pass sequences, demonstrating an effect of spatial entropy when the sequences were matched in contrast. As predicted, the low-pass and fixed sequences dominated a comparable proportion of the total viewing time, indicating a null effect of temporal entropy. (b) Mean dominance duration results. Similar to predominance, the standard sequence had higher mean dominance durations than the low-pass and fixed sequences, and there was no significant difference between the fixed and sine sequences.

The predominance and mean dominance duration results of each match are summarised in Figure 5. As in the previous experiments, individual data points representing the average predominance and mean dominance duration for each participant are shown for all three matches. Figure 5(a) reveals that the standard Mondrian sequence dominated perception for a larger proportion of the viewing time than the low-pass and fixed sequences, that is, 14% and 30% higher in median predominance, respectively. On the other hand, when low-pass and fixed sequences were pitted against each other, both sequences dominated comparable proportions of the viewing time (approximately 40% to 50% each). Similar trends were observed with mean dominance durations (Figure 5(b)), and our observations corroborated with the statistical results. Compared with the low-pass and fixed sequences, the standard sequence had a significantly longer predominance, *V* = 28, *p* = .02 for both, and a significantly larger mean dominance duration, *V* = 28, *p* = .02 and *V = *28*, p* = .03, respectively. No significant differences were observed between low-pass and fixed sequences for predominance, *V* = 8, *p* = .34, and mean dominance duration, *V* = 15, *p* = .94. Supplementary analyses showed that the standard sequence had a comparable advantage over fixed and low-pass sequences. That is, the difference in predominance between the standard and fixed sequences was statistically comparable between that of the standard and low-pass sequences, *V* = 19, *p* = .47, and the same trend was observed in mean dominance durations, *V* = 11, *p* = .69.

**Figure 6.**
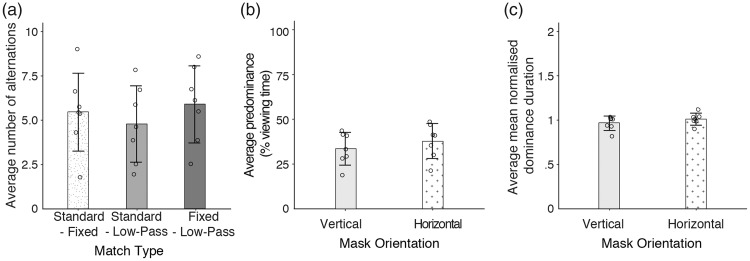
Results for alternation frequency and orientation in Experiment 5. (a) Alternation frequency for each boxing match. The number of perceptual switches was comparable across all three matches, demonstrating that spatial entropy in the standard sequence did not trigger more perceptual switches but prolonged mean dominance and predominance (cf. Figure 5). (b) Predominance of vertically and horizontally oriented pattern sequences. Horizontally oriented patterns were slightly more dominant than vertically oriented sequences; however, the effect was subtle and not statistically significant. (c) Mean dominance duration of vertically and horizontally oriented sequences. Similar to predominance, both orientations had statistically comparable mean dominance durations. The bar graphs illustrated the distributions of data points, the median and the median absolute deviation (denoted by error bars).

Figure 6 summarises the alternation frequencies of each match, the predominance and mean dominance of horizontally and vertically oriented patterns. As shown in Figure 6(a), the match between fixed and low-pass sequences had approximately 20% lower number of alternations than the other two matches. Collapsed across all three matches, we found that predominance and mean dominance were comparable between vertically and horizontally oriented patterns. Statistical analyses showed that the differences in alternation frequencies were not significant, χ^2^(2) = 4.6, *p* = .10. The predominance and mean dominance duration of vertically and horizontally patterns were also not significantly different, *V* = 5*, p = *.16 and *V* = 10, *p* = .58, respectively.

### Discussion for Experiment 5

Experiments 3 and 4 revealed a modest influence of spatial entropy that was only observed with stepped presentation schedules. As stepped presentation schedules have a broadband temporal frequency spectrum, we asked if the effect of spatial entropy was dependent on the presence of higher temporal frequency content. In addition, because Experiments 3 and 4 had used a large contrast imbalance between the eyes, we questioned if the effect of entropy was limited by the existing strong suppression of the target. To investigate these questions, we eliminated the contrast imbalance between the eyes in Experiment 5 and pitted standard, fixed and low-pass Mondrian sequences against each other in a rivalry paradigm (or boxing match). Despite the removal of the interocular contrast imbalance, we obtained similar results to Experiments 3 and 4. Specifically, the standard sequence produced significantly higher predominance and mean dominance durations than the fixed sequence. This advantage, however, was not observed when low-pass and standard sequences rivalled against each other. The low-pass sequence was less dominant than the standard sequence, but it performed comparably to the fixed sequence. Since spatial entropy was preserved in the low-pass sequence, the advantage obtained with spatial entropy was dependent on the presence of the low energy, high temporal frequency content in the standard masker. We also found no significant differences when we compared the alternation rates of the different matches. On some other tasks not involving interocular competition, sensitivity differences between horizontal and vertical orientations have been found (e.g., length judgments: Gottsdanker & Tietz, 1992), but we saw no evidence that those orthogonal orientations differed in predominance during binocular rivalry. Thus, the main effect of spatial entropy was to prolong each period of suppression throughout the course of the viewing time. In addition, this effect was dependent on the higher temporal frequency content and not likely to be a consequence of pattern orientation.

Compared with temporally high-pass filtered Mondrian maskers, Han et al. (2018) found that CFS suppression was stronger when temporally low-pass filtered Mondrian maskers contained coherent pattern edges. While these findings argued for a pairing of low temporal frequencies and pattern edges in CFS, note that the study examined suppressive effectiveness using isolated high and low temporal frequency content. By keeping the temporal frequency content of our Mondrian sequences predominantly biased towards low frequencies, our results showed that the low energy, higher temporal frequencies in the Mondrian masker could enhance interocular suppression but only if they were paired with spatial entropy. Without spatial entropy, higher temporal frequencies in the fixed sequence did not provide any advantage over the low-pass sequence presumably because of increased adaptation (see Alais et al., 2010) and inhibitory action from the higher frequencies to the lower frequencies (Cass & Alais, 2006). As spatial entropy only provided an advantage when transients were present (cf. Figure 5(b)), our results hinted at the presence of pattern structure masking processes (see Enns & Di Lollo, 2000; Macknik et al., 2000) which might act in tandem with lower level rivalry processes suggested elsewhere (e.g., Han et al., 2016; Yang & Blake, 2012).

## Conclusion

One of the important concepts in the early years of cognitive science was the notion of information entropy. Originating in data communication theory (Shannon, 1948), entropy expresses the simple idea that the predictability of an event is inversely related to its information content. Expressed even more colloquially, surprising outcomes convey more information than expected ones (Friston, 2018). We have applied this notion to the analysis of monocularly viewed sequences of rapidly presented arrays of image features forming animations used to induce a potent form of interocular suppression dubbed CFS (Tsuchiya & Koch, 2005). In particular, we have varied the informational uncertainty, that is, the entropy, of CFS sequences in both the space and time domains, in an effort to identify the conditions favouring maximal suppression strength.

Our results show that temporal entropy has little influence on CFS effectiveness. This is perhaps not surprising, as previous work has implicated low temporal rates as optimal for CFS effectiveness (Han et al., 2016, 2018), and slow oscillations are predictable and thus low in entropy. Increasing the Mondrian refresh rate, however, has been shown to broaden the temporal tuning function of CFS (Tsuchiya & Koch, 2005; Zhu et al., 2016). Had temporal entropy been a dominant part of CFS effectiveness, we would expect suppression strength to rise with refresh rate because higher refresh rates broaden the temporal frequency spectrum and increase its resemblance to white noise. Spatially, we observed measurable, but modest effects of predictability. The presence of unpredictable pattern changes, that is, higher entropy, prolonged masker predominance significantly, but only if the maskers compared had stepped presentation schedules and were also presented with a temporally compatible target (Figure 4(a)). Pitting the different Mondrian sequences in a rivalry paradigm showed that the effect of spatial entropy was dependent on the presence of the low energy, higher temporal frequency content in the Mondrian.

These results demonstrate some interesting points. First, studies have reported effects of feature selectivity in interocular suppression (Han & Alais, 2018; Moors et al., 2014; Stuit, Cass, Paffen, & Alais, 2009). Although we did not test low-level properties explicitly, we did obtain evidence of temporal frequency selectivity. For example, spatial entropy only had a significant effect on CFS dynamics when the target and masker were temporally compatible (Figure 4(a)). Similarly, manipulating the regularity of pattern updates did not result in significant differences in CFS dynamics, presumably because of the comparable temporal frequency content between the regular and irregular pattern update schedules (Figures 2 and 3(a)). Second, temporal frequency selectivity was not the only low-level influence in our data, as fixed and sine sequences performed comparably regardless of the target rate (Figure 4). Apart from the similarities between the 1/f temporal spectrum and slow sine modulation (both have low frequency power in common), other factors include the stepped presentation and high temporal frequency content of the fixed sequence. These factors could promote neural adaptation (see Alais et al., 2010) and cross-channel inhibition on lower frequencies (Cass & Alais, 2006), weakening any potential advantage of the broader frequency spectrum of the fixed masker.

This brings us to the third point. If the low-level factors associated with the stepped presentation of the fixed masker were detrimental to its effectiveness, how were these limitations overcome by spatial entropy in the standard masker? We surmise that the unpredictable, dynamic edges in the Mondrian pattern comprise the key feature of the spatial entropy effect, as spatially uncorrelated and unpredictable noise patterns have been shown to be weaker than intact Mondrian patterns (Drewes, Zhu, & Melcher, 2018; Han et al., 2018). Since the effect of spatial entropy was dependent on the presence of higher temporal frequencies in Experiment 5, we postulate that the transient, unpredictable phase congruencies in the Mondrian may provide some form of pattern masking (see Enns & Di Lollo, 2000; Macknik et al., 2000). As has been suggested by previous reports (Han et al., 2018; Tsuchiya & Koch, 2005; Yang & Blake, 2012), the masking processes could further enhance ongoing rivalry suppression processes in CFS. Based on our current results and a previous report by Han et al. (2018), the extent of masking influence appears to be small, though it may become useful for impairing target visibility at later phases of CFS suppression. Our results in Experiment 3 (Figure 4(a)) also suggest that masking could aid in maintaining the effectiveness of CFS suppression over prolonged periods.

In closing, we should stress that our analysis is based on measures of CFS effectiveness collected over extended viewing periods of 60s (Experiments 1–4) or 90s (Experiment 5). In our task, participants tracked the changes in visibility and suppression of the target, similar to the common *dominance tracking* task used in binocular rivalry to quantify alternation dynamics (e.g., Brascamp et al., 2006), but seldom used in CFS. It is possible that different perspectives on CFS strength might arise if suppression potency were measured using another approach, such as measuring the initial suppression duration before the target breaks through to visibility (the so-called b-CFS method; e.g., Jiang et al., 2007). Another useful approach would be to measure the transient reduction in visual sensitivity of the suppressed eye using the contrast probe technique, as often used in binocular rivalry to measure the strength of suppression (Nguyen, Freeman, & Alais, 2003) and occasionally used in CFS studies (Tsuchiya, Koch, Gilroy, & Blake, 2006), although recent work suggests these measures are highly correlated (Han & Alais, 2018, see Figures 2 and 3).

## Supplemental Material

sj-vid-1-ipe-10.1177 2041669518792930 - Supplemental material for Battle of the Mondrians: Investigating the Role of Unpredictability in Continuous Flash Suppression Click here for additional data file. Supplemental material, sj-vid-1-ipe-10.1177 2041669518792930 for Battle of the Mondrians: Investigating the Role of Unpredictability in Continuous Flash Suppression by Shui’Er Han, David Alais and Randolph Blake in i-Perception

## Supplemental Material

sj-vid-2-ipe-10.1177 2041669518792930 - Supplemental material for Battle of the Mondrians: Investigating the Role of Unpredictability in Continuous Flash Suppression Click here for additional data file. Supplemental material, sj-vid-2-ipe-10.1177 2041669518792930 for Battle of the Mondrians: Investigating the Role of Unpredictability in Continuous Flash Suppression by Shui’Er Han, David Alais and Randolph Blake in i-Perception

## Supplemental Material

sj-vid-3-ipe-10.1177 2041669518792930 - Supplemental material for Battle of the Mondrians: Investigating the Role of Unpredictability in Continuous Flash Suppression Click here for additional data file. Supplemental material, sj-vid-3-ipe-10.1177 2041669518792930 for Battle of the Mondrians: Investigating the Role of Unpredictability in Continuous Flash Suppression by Shui’Er Han, David Alais and Randolph Blake in i-Perception
